# Relationship between vitamin D deficiency and gestational diabetes: a narrative review

**DOI:** 10.3389/fendo.2024.1504930

**Published:** 2024-12-19

**Authors:** Caiqiong Lin, Haiwei Liu

**Affiliations:** Department of Endocrinology, Hainan General Hospital, Hainan Affiliated Hospital of Hainan Medical University, Haikou, Hainan, China

**Keywords:** vitamin D deficiency, vitamin D receptor gene, gestational diabetes mellitus, correlation, dose supplementation

## Abstract

Vitamin D, often referred to as the “sunshine vitamin,” is an essential fat-soluble vitamin that plays a critical role in bone health and has been shown to improve insulin sensitivity and glucose tolerance. Vitamin D deficiency is prevalent among pregnant and pre-pregnancy women, which increases the risk of developing gestational diabetes mellitus (GDM), a common complication during pregnancy. Recent studies have explored various aspects of the relationship between vitamin D deficiency and GDM, including the mechanisms by which vitamin D affects glucose metabolism, the role of the vitamin D receptor gene, and the impact of routine vitamin D supplementation before and during pregnancy. This paper will review the current research progress in these areas.

## 1 Introduction

GDM refers to abnormal glucose metabolism that occurs during pregnancy, excluding pre-existing type 1 or type 2 diabetes (1, 2). The prevalence of gestational hyperglycemia in China is significant and continues to rise annually. According to the 9th edition of the International Diabetes Federation Diabetes Atlas, the estimated prevalence of GDM in China in 2019 and beyond is 14.8% (3). The 10th edition of the Global Diabetes Map indicates that the global incidence of gestational hyperglycemia in 2021 is 16.7%, of which GDM accounts for 80% (4). the average prevalence of GDM in China is 14.8% (5). Although the etiology and pathogenesis of GDM are not fully understood, several high-risk factors contribute to its increased incidence. Vitamin D is an essential nutrient obtained from sunlight, natural foods, and exogenous supplements (6). Vitamin D_3_, in particular, is found in animal-based foods such as milk, deep-sea fish, cod liver oil, and egg yolk (7).The primary physiological functions of vitamin D include regulating serum calcium absorption, balancing calcium and phosphorus metabolism, promoting bone growth, and regulating cellular growth and differentiation. Vitamin D deficiency in women of childbearing age has also attracted considerable attention. It poses a major health risk not only to non-pregnant women but also to those who are pregnant. Vitamin D is crucial for women of childbearing age and during pregnancy. Deficiency in vitamin D has been shown to affect glucose metabolism mechanisms during pregnancy, including insulin secretion and resistance. This deficiency exacerbates insulin resistance, leading to elevated blood glucose levels and increasing the risk of developing GDM. Furthermore, vitamin D deficiency significantly impacts adverse pregnancy outcomes in women with GDM. Currently, the mechanisms of glucose metabolism during pregnancy, the role of the vitamin D receptor (VDR) gene in GDM, and dose-related indicators of GDM require further research. This article reviews the correlation between vitamin D deficiency and GDM for clinical reference.

## 2 Overview of vitamin D deficiency

### 2.1 Sources of vitamin D

**Figure 1:**
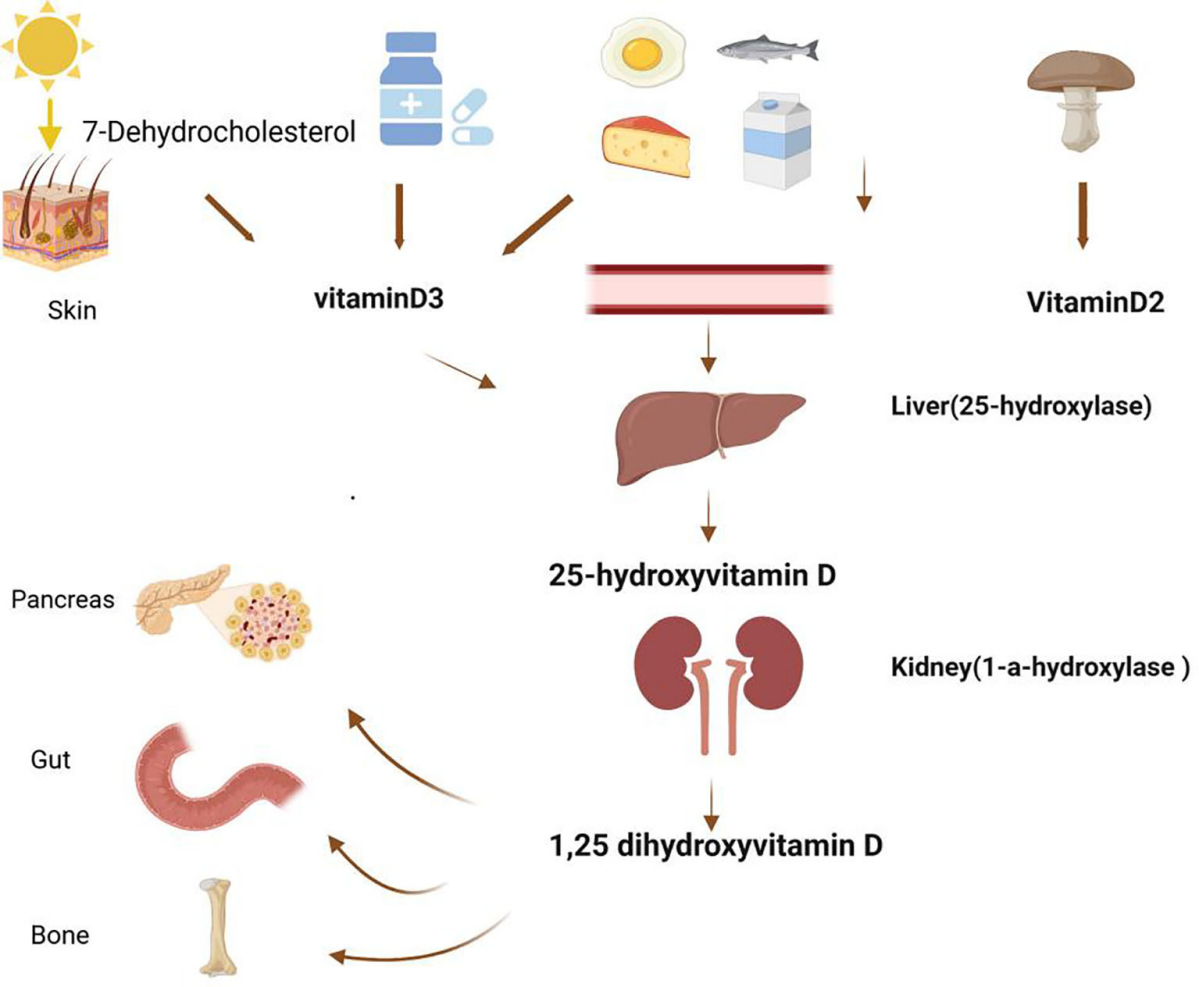
Metabolic pathways of vitamin D.

Vitamin D is a steroid-derived compound obtained from sunlight, natural foods, and exogenous supplements (6). It is mainly acquired through the following methods (**Figure 1**): Sunlight Exposure: The primary source of vitamin D for the human body is through skin exposure to sunlight. When the skin is exposed to ultraviolet B (UVB) radiation from sunlight, 7-dehydrocholesterol in the skin is converted to provitamin D_3_, which then undergoes spontaneous isomerization to form vitamin D3. This process accounts for approximately 80-90% of the body’s vitamin D supply. Food Intake: Vitamin D can also be ingested through dietary sources. Vitamin D_3_ is predominantly found in animal-based foods, such as milk, deep-sea fish, cod liver oil, and egg yolk (7). Vitamin D_2_, on the other hand, mainly comes from plant-based foods like certain mushrooms. Once consumed, vitamin D from these foods enters the lymphatic system via chylomicrons and eventually reaches the bloodstream. Supplement Intake: Due to the limited amount of vitamin D available in food and the possibility of insufficient sunlight exposure, exogenous supplements are an important alternative source of vitamin D. Vitamin D metabolism involves several key steps. In the liver, both vitamin D_3_ and D_2_ are converted to 25-hydroxyvitamin D, the main circulating form of vitamin D and an indicator of vitamin D levels. In the kidneys or extrarenal tissues, 25(OH)D_3_ is further converted to 1,25(OH)_2_D_3_, the active form of vitamin D. This active form is crucial for maintaining calcium and phosphorus balance, bone health, and various other physiological functions.Ensuring adequate vitamin D levels is essential for overall health. In addition to sufficient sunlight exposure, increasing the intake of vitamin D-rich foods can help maintain these levels. However, because dietary sources alone are often inadequate to meet the body’s needs, vitamin D is uniquely referred to as the “sunshine vitamin.”

### 2.2 The harm of vitamin D deficiency

Vitamin D deficiency is potentially harmful to the development and progression of various diseases, with low concentrations of 25(OH)D_3_ serving as potential risk markers for several conditions, including cancer morbidity and mortality. The most well-recognized role of vitamin D is its impact on bone health. A deficiency in vitamin D can lead to inadequate calcium absorption, and severe deficiency may result in bone health diseases. Cancer: Vitamin D has been implicated in the development of cancers such as colon and breast cancer. A meta-analysis of prospective studies assessing the association between serum 25(OH)D_3_ levels and cancer incidence (8 studies) or cancer mortality (16 studies) found that each 20 nmol/L increase in serum 25(OH)D_3_ levels (8 μg/L) was associated with a 7% reduction in cancer risk and a 2% reduction in cancer mortality (8). Cardiovascular Disease: A meta-analysis of prospective studies found an association between reduced vitamin D status, as measured by serum 25(OH)D_3_ levels or vitamin D intake, and an increased risk of ischemic stroke and ischemic heart disease (9). Endocrine and Metabolic Diseases: Studies have indicated that vitamin D deficiency may be closely related to an increased risk of diabetes and pre-diabetes (10). Autoimmune Diseases: Research has shown that vitamin D supplementation can reduce the risk of autoimmune diseases by 22%, and long-term vitamin D supplementation can help prevent these diseases, particularly in individuals aged 50 years and older (11). Other Related Diseases: There is growing evidence that vitamin D deficiency is associated with an increased risk of acute respiratory and chronic diseases, including chronic kidney disease, neurological diseases, and metabolic syndrome. Several studies support the hypothesis that low levels of serum 25(OH)D_3_ are independently associated with the incidence and severity of respiratory tract infections in both children and adults (12, 13). Therefore, it is important to address the potential harm caused by vitamin D deficiency and reduce the incidence of systemic diseases related to it.

### 2.3 Potential mechanisms of vitamin D deficiency on glucose metabolism during pregnancy

#### 2.3.1 Effect of vitamin D deficiency during pregnancy on insulin secretion

Vitamin D deficiency during pregnancy may impact insulin secretion. Over the past five years, numerous studies have highlighted vitamin D’s crucial role in both insulin secretion and insulin resistance. Vitamin D can regulate insulin secretion from pancreatic β-cells by altering the expression of the proinsulin gene. Studies have shown that 1,25(OH)_2_D_3_ enhances calcium influx during glucose-stimulated insulin secretion (GSIS) by up-regulating related genes, thereby modulating beta cell insulin secretion (14). Additionally, the interaction between vitamin D and the vitamin D receptor (VDR) on pancreatic β-cells can regulate extracellular calcium concentration and calcium flux through ion channels. This process facilitates calcium-dependent insulin secretion via the calcium concentration gradient across the cell membrane, promoting insulin release. L-type voltage-gated calcium channels (L-VGCC), K^+^-ATP, and K^+^-Ca^2+^ channels are involved in 1,25(OH)_2_D_3_ signaling. Transcriptional regulation of voltage-gated calcium channels by 1,25(OH)_2_D_3_ through VDR also influences GSIS (14–16). Animal studies have shown that 1,25(OH)_2_D_3_ can stimulate insulin secretion in a sugar-independent manner, promoting islet insulin release (16). Bornstedt Mette Eskild found a significant increase in insulin secretion in cells treated with 1,25(OH)_2_D_3_, suggesting that vitamin D enhances GSIS (17). This effect has also been observed in human islets. Conversely, vitamin D deficiency may reduce calcium ion concentration in islet cells, impairing related signaling pathways and affecting insulin synthesis and secretion, leading to elevated blood glucose levels and potentially resulting in GDM.

#### 2.3.2 Effect of vitamin D deficiency during pregnancy on insulin resistance

##### 2.3.2.1 Vitamin D deficiency during pregnancy reduces insulin receptor expression

Vitamin D indirectly affects insulin secretion by reducing inflammatory responses and improving insulin resistance (18). Research has verified that 1,25(OH)_2_D_3_ can improve insulin resistance (IR) in trophoblast cells by inhibiting the mTOR signaling pathway, as demonstrated through the establishment of an IR BeWo cell model. 1,25(OH)_2_D_3_ protects trophoblasts from high IR primarily by inhibiting mTOR signaling, which may be a potential therapeutic approach for patients with GDM (19).During pregnancy, vitamin D deficiency leads to reduced levels of 1,25(OH)_2_D_3_, which diminishes the inhibition of the mTOR signaling pathway, resulting in increased insulin resistance and a higher incidence of GDM.

##### 2.3.2.2 Vitamin D deficiency during pregnancy exacerbates inflammation and oxidative response

Vitamin D plays a crucial role in both the inflammatory response and oxidative stress. Vitamin D, by binding with its receptor, reduces pro-inflammatory cytokines in immune cells and has an immunomodulatory effect (20, 21). Studies have shown that treatment with 1,25(OH)_2_D_3_ in GDM placental explants blocks the abnormal increase in leptin, tumor necrosis factor-alpha (TNF-α), and interleukin-6 (IL-6) levels, reducing both placental IR and inflammatory responses (19). This suggests that 1,25(OH)_2_D_3_ is involved in maintaining normal immune inflammatory responses, especially during pregnancy when CYP27B1 is strongly expressed in the placenta, becoming an important source of 1,25(OH)_2_D_3_ synthesis (22). Furthermore, low vitamin D levels not only exacerbate systemic inflammation but also promote placental inflammation (23).

##### 2.3.2.3 Vitamin D deficiency and obesity during pregnancy increase insulin resistance

Obesity is characterized by body mass index (BMI) greater than 30, while a BMI greater than 25 shows that the individual is overweight (24). Several studies have shown that vitamin D deficiency is strongly associated with insulin resistance, especially in obesity and in patients with metabolic syndrome (25, 26). Several studies have shown that low levels of vitamin D are strongly associated with the development of insulin resistance, especially in obese and type 2 diabetic patients (27, 28). 1,25(OH)_2_D_3_ can regulate adipocyte formation and differentiation by modulating the nuclear receptor VDR and peroxisome proliferator-activated receptor γ (PPARγ) pathways. It has been reported that the serum vitamin D levels in women with GDM and those who are overweight or obese are reduced, while the expression of VDR and PPARγ mRNA in adipose tissue is up-regulated (29). This up-regulation further increases the expression in overweight or obese women with GDM and contributes to the development of GDM. Some scholars found that pregnant women with a pre-pregnancy BMI of 23.5-27.0 kg/m² could significantly reduce the risk of GDM by increasing their serum vitamin D levels, suggesting a synergistic effect between low vitamin D levels and obesity (30). Research has confirmed that vitamin D deficiency is strongly associated with obesity (25). Further studies have indicated that low serum 25OHD is positively correlated with obesity or BMI in adults and children, and vitamin D plays an important role in adipogenesis and inflammation of adipocytes and adipose tissue (31). These findings suggest that vitamin D deficiency promotes obesity by enhancing the expression of the PPARγ pathway, thereby regulating the development and differentiation of adipocytes. Vitamin D supplementation may become a nutritional intervention for GDM, with significant clinical implications for reducing the incidence of GDM, particularly in obese or overweight women.

### 2.4 The relationship between vitamin D level and GDM in women before pregnancy

#### 2.4.1 Vitamin D deficiency in non-pregnant women of childbearing age

Due to lifestyle changes and environmental factors, vitamin D deficiency has become a common problem, especially for women of childbearing age. Research investigating serum 25(OH)D_3_ levels in Chinese women of gestational age from cities between 2010 and 2012 found that only 15.1% had normal vitamin D nutritional status (32). This indicates that women of childbearing age often overlook the significant health issues caused by vitamin D deficiency. A prospective cohort study showed that vitamin D deficiency in women of childbearing age can adversely affect the female reproductive system, leading to infertility (33). Furthermore, studies have demonstrated that in the polycystic ovary syndrome(PCOS) population, vitamin D deficiency has a higher prevalence of glucose intolerance than women without vitamin D deficiency (34). The study by Wehr E provides compelling evidence that women with normal ovulation have higher vitamin D levels than women with PCOS (35). A recent review by Iervolino et al. Concluded that vitamin D appears to be effective in the treatment of PCOS (36). Additionally, Di Bari noted an association between low 25(OH)D_3_ levels and obesity, hyperandrogenism, insulin resistance, and other metabolic dysfunctions associated with PCOS (37). These studies highlight the importance of vitamin D intake and supplementation for women of childbearing age. Regular examination of 25(OH)D_3_ levels should be considered a routine part of physical examinations for young women and before pregnancy. Regular assessment of 25(OH)D_3_ levels can help to monitor vitamin D status and guide the appropriate dosage of supplements. By actively maintaining adequate vitamin D levels, women of childbearing age can better protect their health.

### 2.5 Routine pre-pregnancy vitamin D supplementation for women of childbearing age

The increasing number of problems caused by vitamin D deficiency has gradually attracted societal attention. While the necessity of routine vitamin D supplementation before pregnancy remains a debated issue, but vitamin D supplementation is extremely necessary. Recent studies have shown that vitamin D is associated with fertility and suggest that optimal levels of 30 ng/mL or higher should be achieved with appropriate doses before and throughout pregnancy (38). It is also essential to continue vitamin D supplementation during pregnancy. Rosalyn J Singleton found that prenatal supplementation with 1000 IU of vitamin D_3_ significantly increased prenatal 25(OH)D concentrations. This increase may help reduce the rate of early childhood caries and provides a reference for prenatal vitamin D supplementation in other high-risk groups for rickets (39). The benefits of routine vitamin D supplementation before pregnancy are evident, though there are currently few studies on this topic. Future research should focus on supplementing different doses of vitamin D according to varying degrees of deficiency, which requires further exploration.

### 2.6 The relationship between vitamin D deficiency and gestational diabetes mellitus

#### 2.6.1 Vitamin D receptor gene and GDM

**Figure 2:**
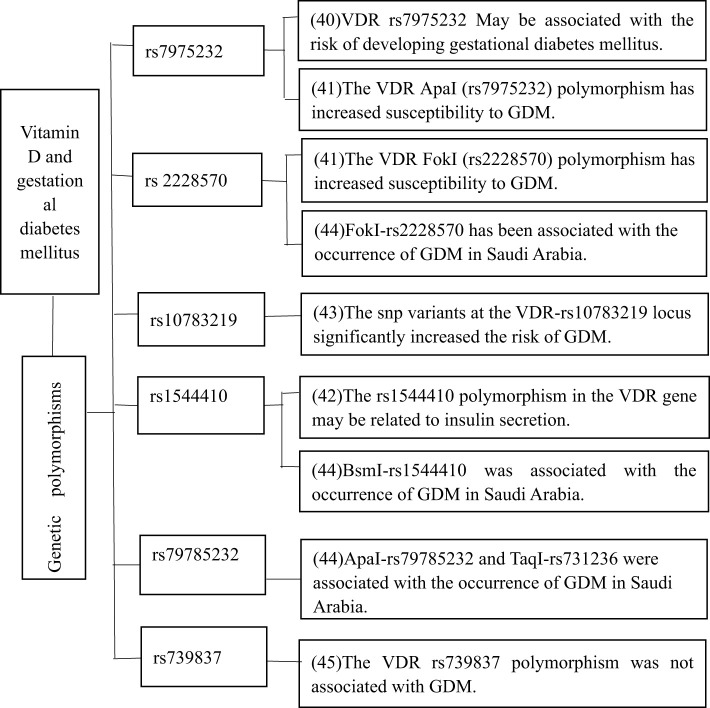
The relationship between vitamin D receptor genes and gestational diabetes mellitus.

The relationship between the VDR gene and GDM has garnered significant attention in recent years. Consequently, polymorphisms in the VDR gene may be linked to an increased risk of GDM. Several studies have demonstrated that VDR gene polymorphism may play a role in the pathogenesis of GDM (**Figure 2**). For instance, polymorphisms at sites such as rs7975232, rs2228570, and rs1544410 have been linked to an elevated risk of GDM, providing insights into how the VDR gene influences the likelihood of developing GDM. Research has shown that the rs7975232 polymorphism in the VDR gene may be associated with GDM risk (40). A meta-analysis by Sai Liu and colleagues supported the association between the VDR rs7975232 polymorphism and GDM, and also found that the FokI (rs2228570) polymorphism was linked to increased susceptibility to GDM (41). Additionally, it has been demonstrated that the rs1544410 polymorphism in the VDR gene is associated with insulin secretion in GDM patients (42). An important study confirmed that single nucleotide polymorphism (SNP) mutations at VDR-rs10783219 and MTNR1B-rs10830962 significantly increase the risk of GDM (43). Further research in Saudi Arabia found that ApaI-rs79785232, BsmI-rs1544410, FokI-rs2228570, and TaqI-rs731236 polymorphisms are related to the occurrence of GDM in the region (44). In conclusion, the VDR gene does play a role in the pathogenesis of GDM. Although most studies support the association between the VDR gene and GDM, a few have not found such a link. It has been reported that the VDR gene rs739837 polymorphism is not associated with GDM (45).

#### 2.6.2 Relationship between vitamin D deficiency and GDM

Vitamin D deficiency is prevalent among pregnant women. A study in Switzerland found that 73.2% of pregnant women had vitamin D deficiency or insufficiency (46). Similarly, research in Boston, USA, revealed that 53.2% of 206 pregnant women had vitamin D levels below 30 ng/mL, indicating that vitamin D deficiency remains widespread and significantly increases the incidence of GDM (47). Maysa Alzaim demonstrated a 1.29-fold increase in the risk of GDM for every 12.5 nmol/L decrease in serum 25(OH)D_3_ levels (48). The Third International Conference on Vitamin D Controversy in 2020 reached an international consensus showing that about 7% of the global population suffers from severe vitamin D deficiency, with prevalence rates of 37% worldwide, 40% in Europe, and 72% in China (49). A review of 36 observational studies found that the risk of GDM in pregnant women with vitamin D deficiency increased by 18%, and serum 25(OH)D_3_ levels in women with GDM were 1.18 nmol/L lower, suggesting a link between low vitamin D concentrations and GDM (50). A retrospective cohort study by Yan Cheng showed that in the vitamin D status of pregnant women in Shanghai and its relationship with GDM, vitamin D deficiency and insufficiency were prevalent among women in Shanghai, and vitamin D levels of at least 20 ng/mL in early pregnancy was significantly associated with reduced risk of GDM (51). It is suggested that high levels of vitamin D have a protective effect on the risk of GDM. A nested case-control study by Eleonora Salakos et al. found that women with25(OH)D_3_ levels below 20 ng/mL had a significantly higher risk of GDM compared to non-GDM patients (52). Furthermore, a prospective cohort study by Alireza Milajerdi showed that individuals with vitamin D deficiency had a 26% higher risk of developing GDM than those with normal serum vitamin D levels (OR: 1.26; 95% CI: 1.13, 1.41). There was a significant positive association between vitamin D insufficiency and deficiency and GDM risk (OR: 1.23; 95% CI: 1.11, 1.35). The study found that the risk of GDM was lowest in individuals with serum vitamin D levels between 40 and 90 nmol/L, and a dose-response analysis revealed a U-shaped nonlinear correlation between serum vitamin D concentration and GDM risk (P < 0.05) (53).

#### 2.6.3 Correlation between vitamin D dose and gestational diabetes mellitus

There are numerous reports about the controversy surrounding vitamin D supplementation for GDM, but vitamin D is generally considered an effective treatment for GDM (**Table 1**). The latest recommendation from the Institute of Medicine (IOM) for vitamin D supplementation during pregnancy and lactation is 600 IU per day (54). In 2011, the Endocrine Society issued guidelines on the assessment, diagnosis, and treatment of vitamin D deficiency, recommending that pregnant and lactating women should receive at least 600 IU of vitamin D per day, with a target 25(OH)D_3_ level of at least 30 ng/mL (55). The Central and Eastern European expert consensus statement recommends that women planning to become pregnant should initiate or maintain vitamin D supplementation, with healthy adults advised to take 800-2000 IU/day if they have no other risk factors. A treatment duration of entire pregnancy and lactation is recommended, with the aim to target concentrations of 30 to 50 ng/mL (59). Qingying Zhang found that high-dose and moderate-dose vitamin D supplementation reduced insulin and HOMA-IR levels in GDM patients. Randomized controlled trials indicated that high-dose vitamin D supplementation (50000 IU every two weeks) significantly reduced insulin resistance in pregnant women with GDM. It is recommended that pregnant women with GDM receive high-dose vitamin D supplementation (50000 IU every two weeks) from the 12th week of gestation until delivery (56). The AME statement from the Italian Association of Clinical Endocrinology suggests that a safe dose of vitamin D supplementation during pregnancy is 4000 IU/day, with a therapeutic target serum 25(OH)D_3_ level of > 40 ng/mL (57). The expert panel, including the Polish Association of Pediatric Endocrinology and Diabetes, recommends a dose of 2000 IU/day for pregnant and lactating women, aiming for a serum level of 30-50 ng/mL, with treatment lasting 12 weeks or until the target concentration is achieved (58). A study by Eduardo Klöppel showed that vitamin D supplementation in pregnant rats was more beneficial than no supplementation, aiding fetal development and reducing prediabetic complications (60). Another study demonstrated that GDM patients who supplemented with vitamin D and omega-3 fatty acids for six weeks experienced significant reductions in fasting blood glucose, triglycerides, high density lipoprotein, Low-density lipoprotein and total cholesterol, ultimately improving glucose and lipid metabolism (61). Therefore, vitamin D supplementation is particularly important, and further research is needed to determine optimal supplementation strategies for different baseline levels of vitamin D deficiency.

**Table 1:** Vitamin D supplementation is recommended for pregnant women.

Country or Region (Year)	Population	Size of Population	Gestational Week (GW)	Oral Vitamin D (IU)	Treatment Duration	Target Concentration (ng/mL)	First Author
Institute of Medicine (2011)	Pregnant and lactating women	/	/	600 IU/day	/	/	ACOG Committee (54)
Endocrine Society (2011) USA	Pregnant and lactating women	/	/	600 IU/day	/	30	Holick et al. (55)
Exp Ther Med (2016)	Gestational diabetes	133	24-28 GW	50000 IU/2weeks	12^th^ week to delivery	/	QINGYING ZHANG et al. (56)
Italy (2018)	Pregnant women	/	/	4000 IU/day	/	>40	Cesareo et al. (57)
Poland (2018)	Pregnant and lactating women	/	/	2000 IU/day	12 weeks	>30–50	Rusińska A et al. (58)
A Central and Eastern European (2022)	BeforePregnant 、Pregnantand lactating women	/	/	800-2000 IU/day	throughout pregnancy and lactation	30-50	Pawel Pludowski et al. (59)

### 2.7 Effect of vitamin D deficiency on the outcome of pregnant women with GDM

Vitamin D plays a crucial role during pregnancy, impacting not only the health of pregnant women but also being closely related to adverse pregnancy outcomes. For instance, vitamin D deficiency has been linked to an increased rate of cesarean sections, GDM and preeclampsia. An increasing number of studies highlight the significant impact of vitamin D deficiency on pregnancy outcomes (**Table 2**). Anne Merewood showed that women with 25(OH)D_3_ levels below 37.5 nmol/L were four times more likely to have a cesarean section compared to those with levels of 37.5 nmol/L or higher, suggesting a negative correlation between vitamin D deficiency and cesarean section rates (62). Another study supported this association, finding that pregnant women in Singapore with insufficient 25(OH)D_3_ levels had a higher likelihood of emergency cesarean section (OR= 1.39, 95% CI = 0.95, 2.05) (69). A prospective cohort study by Hanna Augustin found that vitamin D deficiency was associated with a two-fold increased risk of emergency cesarean section in women without epidural anesthesia (64). Similarly, Mina Amiri found that women with moderate vitamin D deficiency were more likely to undergo cesarean section. Severe vitamin D deficiency exhibited a higher probability of preterm delivery, indicating that vitamin D status at delivery can directly affect the mode of delivery (68). However, studies have been inconsistent regarding the association between vitamin D levels and pregnancy outcomes. Some research has found no association between maternal vitamin D levels and the risk of vaginal birth, instrumental delivery, primary cesarean delivery, or cesarean delivery for any other reason (70). Similarly, other studies reported that vitamin D deficiency In women with GDM at mid-pregnancy is associated with an elevated risk of postpartum glucose intolerance (71). Premature rupture of membranes (PPROM) is another adverse pregnancy outcome linked to vitamin D deficiency. A prospective study by Hyun Joo Lee measured vitamin D levels in 355 pregnant women during the first trimester and before delivery, finding that the incidence of PPROM was higher in the vitamin D deficiency group compared to the non-deficiency group. Vitamin D levels were significantly lower in the PPROM group during both the first and second trimesters, indicating a significant association between vitamin D deficiency and PPROM (p = 0.003) (72). A logistic regression analysis of 2074 pregnant women found that those with severe vitamin D deficiency had an increased risk of preeclampsia (OR 2.08; 95% CI, 1.05-4.13) but the association was rendered non-significant after correction (OR 1.88; 95% CI 0.79-4.48) (63). A study by Juhi Nema reported that continuous measurement of vitamin D throughout pregnancy and the risk of preeclampsia in an Indian population, suggesting that vitamin D deficiency could be an important etiological factor in the clinical diagnosis of preeclampsia (67). Another study found that vitamin D levels were inversely related to the severity of preeclampsia, and the severity of preeclampsia increased with the decrease of vitamin D levels (p < 0.001) (65). Additionally, Shu Qin Wei also indicated maternal vitamin D deficiency was associated with the risk of preeclampsia at 24-26 weeks of gestation (66). Therefore, it is essential to address the negative effects of vitamin D deficiency on pregnancy outcomes, particularly in women with GDM.

**Table 2:** Effect of vitamin D deficiency on adverse outcomes in pregnant women with GDM.

First Author (Year)	Study design	Place of study	Sample size	VitD Assay method	Outcome analyzed	Statistics (95% CI or AOR)	Sample (Serum or Plasma)
Anne Merewood, et al. (2009) (62)	Prospective cohort study	Boston, Massachusetts	253	Liquid chromatography-mass spectrometer	Primary Cesarean Section	AOR = 3.84; 95%CI(1.71-8.62)	Serum
Van Weert et al. (2016) (63)	Prospective cohort study	Netherlands	2074	Enzyme-linked immunosorbent assay	Pregnancy related hypertensive disorders	OR:1.88 (0.79-4.48)	Serum
Hanna Augustin et al. (2020) (64)	Prospective cohort study	Sweden	1832	Liquid chromatography-mass spectrometer	Emergency Caesarean Section	AOR = 2.01 p = 0.044	Serum
Bhupali Das et al. (2021) (65)	case-control study	Indian	1000cases and 1000 controls	Radioimmunoassay	preeclampsia	OR:11.308; 95%CI (7.5982-14.0097)	Serum
Shu Qin Wei, et al. (2021) (66)	Nested case– control study	/	34:65	/	pre-eclampsia	AOR=4.79; 95%CI (1.67-13.75)	plasma
Juhi Nema, et al. (2023) (67)	Longitudinal study	Pune, India.	108cases and 216controls	Enzyme-linked immunosorbent assay	preeclampsia	95% CI (0.08,0.77)	Serum
Mina Amiri, et al. (2023) (68)	Stratified randomized controlled field trial	Khuzestan	1649	Enzyme-linked immunosorbent assay method and a kit of Immunodiagnostics systems	preterm delivery、cesarean section	(95% CI: 25.69–30.02), (95% CI: 33.36–37.96)	Serum

## 3 Conclusion and prospects.

Vitamin D deficiency is very common in pregnant women. With the increasing number of GDM patients worldwide, it is important to pay attention to the negative impact of vitamin D deficiency on pregnant women with GDM. Vitamin D deficiency is also associated with the occurrence of many diseases. Currently, there are numerous conclusions about the potential mechanisms of vitamin D in glucose metabolism and the relationship between the VDR gene and GDM. However, there are still varying results regarding the correlation between vitamin D deficiency and GDM, as well as the treatment and outcomes of vitamin D supplementation for GDM. Future studies should focus on vitamin D supplementation at different levels of deficiency. It is recommended to appropriately supplement vitamin D before and during pregnancy, strengthen the detection of serum 25(OH)D_3_ levels before pregnancy, and achieve early detection and early intervention. This approach can help reduce the impact of vitamin D deficiency on adverse pregnancy outcomes in pregnant women with GDM.

## References

[1] American Diabetes Association Professional Practice Committee. 15. Management of diabetes in pregnancy: standards of medical care in diabetes-2022. Diabetes Care. (2022) 45:S232–43. doi: 10.2337/dc22-S015 34964864

[2] Hod M Kapur A Sacks DA Hadar E Agarwal M Di Renzo GC. The International Federation of Gynecology and Obstetrics (FIGO) Initiative on gestational diabetes mellitus: A pragmatic guide for diagnosis, management, and care. Int J Gynaecol Obstet. (2015) 131 Suppl3:S173–211. doi: 10.1016/S0020-7292(15)30033-3 26433807

[3] Yuen L Saeedi P Riaz M Karuranga S Divakar H Levitt N. Projections of the prevalence of hyperglycaemia in pregnancy in 2019 and beyond: Results from the International Diabetes Federation Diabetes Atlas, 9th edition. Diabetes Res Clin Pract. (2019) 157:107841. doi: 10.1016/j.diabres.2019.107841 31518656

[4] Tönnies T Rathmann W Hoyer A Brinks R Kuss O. Quantifying the underestimation of projected global diabetes prevalence by the International Diabetes Federation (IDF) Diabetes Atlas. BMJ Open Diabetes Res Care. (2021) 9:e002122. doi: 10.1136/bmjdrc-2021-002122 PMC8370495 34400463

[5] Juan J Yang H. Prevalence, prevention, and lifestyle intervention of gestational diabetes mellitus in China. Int J Environ Res Public Health. (2020) 17:9517. doi: 10.3390/ijerph17249517 33353136 PMC7766930

[6] Heaney RP Armas LA. Quantifying the vitamin D economy. Nutr Rev. (2015) 73:51–67. doi: 10.1093/nutrit/nuu004 26024057

[7] Atkinson SA. The new dietary reference intakes from the Institute of Medicine for calcium and vitamin D. Perspect Infirm. (2011) 8 (5):5. 21939083

[8] Han J Guo X Yu X Liu S Cui X Zhang B. 25-hydroxyvitamin D and total cancer incidence and mortality: A meta-analysis of prospective cohort studies. Nutrients. (2019) 11:2295. doi: 10.3390/nu11102295 31561503 PMC6835972

[9] Zhou R Wang M Huang H Li W Hu Y Wu T. Lower vitamin D status is associated with an increased risk of ischemic stroke: A systematic review and meta-analysis. Nutrients. (2018) 10:277. doi: 10.3390/nu10030277 29495586 PMC5872695

[10] McCarthy K Laird E O'Halloran AM Walsh C Healy M Fitzpatrick AL. Association between vitamin D deficiency and the risk of prevalent type 2 diabetes and incident prediabetes: A prospective cohort study using data from The Irish Longitudinal Study on Ageing (TILDA). EClinicalMedicine. (2022) 53:101654. doi: 10.1016/j.eclinm.2022.101654 36147626 PMC9486023

[11] Hahn J Cook NR Alexander EK Friedman S Walter J Bubes V. Vitamin D and marine omega 3 fatty acid supplementation and incident autoimmune disease: VITAL randomized controlled trial. BMJ. (2022) 376:e066452. doi: 10.1136/bmj-2021-066452 35082139 PMC8791065

[12] Gunville CF Mourani PM Ginde AA. The role of vitamin D in prevention and treatment of infection. Inflamm Allergy Drug Targets. (2013) 12:239–45. doi: 10.2174/18715281113129990046 PMC3756814 23782205

[13] Ingham TR Jones B Camargo CA Kirman J. Association of vitamin D deficiency with severity of acute respiratory infection: A case-control study in New Zealand children. Eur Respir J. (2014) 44:439. doi: 10.13140/2.1.3250.0485

[14] Kjalarsdottir L Tersey SA Vishwanath M Chuang JC Posner BA Mirmira RG. 1,25-Dihydroxyvitamin D3 enhances glucose-stimulated insulin secretion in mouse and human islets: a role for transcriptional regulation of voltage-gated calcium channels by the vitamin D receptor. J Steroid Biochem Mol Biol. (2019) 185:17–26. doi: 10.1016/j.jsbmb.2018.07.004 30071248

[15] Alvarez JA Ashraf A. Role of vitamin d in insulin secretion and insulin sensitivity for glucose homeostasis. Int J Endocrinol. (2010) 2010:351385. doi: 10.1155/2010/351385 20011094 PMC2778451

[16] Mendes AKB Sulis PM Cavalari FC Padilla DPR Aragón M Gaspar JM. 1α,25-(OH)2 vitamin D3 prevents insulin resistance and regulates coordinated exocytosis and insulin secretion. J Nutr Biochem. (2022) 99:108864. doi: 10.1016/j.jnutbio 34606907

[17] Bornstedt ME Gjerlaugsen N Pepaj M Bredahl MKL Thorsby PM. Vitamin D increases glucose stimulated insulin secretion from insulin producing beta cells(INS1E). Int J Endocrinol Metab. (2019) 17:e74255. doi: 10.5812/ijem.74255 30881469 PMC6408731

[18] Wu J Atkins A Downes M Wei Z. Vitamin D in diabetes: uncovering the sunshine hormone's role in glucose metabolism and beyond. Nutrients. (2023) 15:1997. doi: 10.3390/nu15081997 37111216 PMC10142687

[19] Li G Lin L Wang YL Yang H. 1,25(OH)2D3 protects trophoblasts against insulin resistance and inflammation via suppressing mTOR signaling. Reprod Sci. (2019) 26:223–32. doi: 10.1177/1933719118766253 29575997

[20] Bishop E L Ismailova A Dimeloe S Hewison M White JH. Vitamin D and immune regulation: antibacterial, antiviral, anti-inflammatory. JBMR Plus. (2020) 5:e10405. doi: 10.1002/jbm4.10405 32904944 PMC7461279

[21] Zughaier SM Lubberts E Bener A. Editorial: immune-modulatory effects of vitamin D. Front Immunol. (2020) 11:596611. doi: 10.3389/fifimmu.2020.596611 33133107 PMC7550665

[22] Park H Wood MR Malysheva OV Jones S Mehta S Brannon PM. Placental vitamin D metabolism and its associations with circulating vitamin D metabolites in pregnant women. Am J Clin Nutr. (2017) 106:1439–48. doi: 10.3945/ajcn.117.153429 PMC5698837 29021285

[23] Zhang Q Chen H Wang Y Zhang C Tang Z Li H. Severe vitamin D deficiency in the first trimester is associated with placental inflammation in high-risk singleton pregnancy. Clin Nutr (Edinburgh Scotland). (2019) 38:1921–6. doi: 10.1016/j.clnu.2018.06.978 30031659

[24] Kuczmarski RJ Flegal KM. Criteria for definition of overweight in transition: background and recommendations for the United States. Am J Clin Nutr. (2000) 72:1074–81. doi: 10.1093/ajcn/72.5.1074 11063431

[25] Greco EA Lenzi A Migliaccio S. Role of hypovitaminosis D in the pathogenesis of obesity-induced insulin resistance. Nutrients. (2019) 11:1506. doi: 10.3390/nu11071506 31266190 PMC6682882

[26] Argano C Mirarchi L Amodeo S Orlando V Torres A Corrao S. The role of vitamin D and its molecular bases in insulin resistance, diabetes, metabolic syndrome, and cardiovascular disease: state of the art. Int J Mol Sci. (2023) 24:15485. doi: 10.3390/ijms242015485 37895163 PMC10607188

[27] Szymczak-Pajor I Śliwińska A. Analysis of association between vitamin D deficiency and insulin resistance. Nutrients. (2019) 11:794. doi: 10.3390/nu11040794 30959886 PMC6520736

[28] Madhu SV Aslam M Mishra BK Gupta A Jhamb R. Association of 25 (OH) vitamin D and leptin in individuals with insulin resistance. Indian J Endocrinol Metab. (2022) 26:435–8. doi: 10.4103/ijem.ijem_141_22 PMC9815200 36618517

[29] Wang HY She GT Sun LZ Lu H Wang YP Miao J. Correlation of serum vitamin D, adipose tissue vitamin D receptor, and peroxisome proliferator-activated receptor γ in women with gestational diabetes mellitus. Chin Med J (Engl). (2019) 132:2612–20. doi: 10.1097/CM9.0000000000000480 PMC6846247 31651513

[30] Yue CY Ying CM. Sufficience serum vitamin D before 20 weeks of pregnancy reduces the risk of gestational diabetes mellitus. Nutr Metab. (2020) 17(1):89. doi: 10.1186/s12986-020-00509-0 PMC7574245 33088335

[31] Ruiz-Ojeda FJ Anguita-Ruiz A Leis R Aguilera CM. Genetic factors and molecular mechanisms of vitamin D and obesity relationship. Ann Nutr Metab. (2018) 73:89–99. doi: 10.1159/000490669 29982250

[32] Lu JX Liu XB Chen J Hu YC Yun CF Li WD. The vitamin D nutritional status in Chinese urban women of child-bearing age from 2010 to 2012. Zhonghua Yu Fang Yi Xue Za Zhi. (2017) 51:112–6. doi: 10.3760/cma.j.issn.0253-9624.2017.02.003 28219147

[33] Pang Y Kim O Choi JA Jung H Kim J Lee H. Vitamin D deficiency and associated factors in south Korean childbearing women: a cross-sectional study. BMC Nurs. (2021) 20:218. doi: 10.1186/s12912-021-00737-6 34724924 PMC8559402

[34] He C Lin Z Robb SW Ezeamama AE. Serum vitamin D levels and polycystic ovary syndrome: A systematic review and meta-analysis. Nutrients. (2015) 7:4555–77. doi: 10.3390/nu7064555 PMC4488802 26061015

[35] Wehr E Pilz S Schweighofer N Giuliani A Kopera D Pieber TR. Association of hypovitaminosis D with metabolic disturbances in polycystic ovary syndrome. Eur J Endocrinol. (2009) 161:575–82. doi: 10.1530/EJE-09-0432 19628650

[36] Iervolino M Lepore E Forte G Laganà AS Buzzaccarini G Unfer V. Natural molecules in the management of polycystic ovary syndrome (PCOS): an analytical review. Nutrients. (2021) 13:1677. doi: 10.3390/nu13051677 34063339 PMC8156462

[37] Di Bari F Catalano A Bellone F Martino G Benvenga S Vitamin D. Bone metabolism, and fracture risk in polycystic ovary syndrome. Metabolites. (2021) 11:116. doi: 10.3390/metabo11020116 33670644 PMC7922814

[38] Mansur JL Oliveri B Giacoia E Fusaro D Costanzo PR. Vitamin D: Before, during and after Pregnancy: Effect on Neonates and Children. Nutrients. (2022) 14:1900. doi: 10.3390/nu14091900 35565867 PMC9105305

[39] Singleton RJ Day GM Thomas TK Klejka JA Desnoyers CA McIntyre MNP. Impact of a prenatal vitamin D supplementation program on vitamin D deficiency, rickets and early childhood caries in an Alaska native population. Nutrients. (2022) 14:3935. doi: 10.3390/nu14193935 36235588 PMC9570803

[40] Aziz A Shah M Siraj S Iqbal W Jan A Khan I. Association of vitamin D deficiency and vitamin D receptor (VDR) gene single-nucleotide polymorphism (rs7975232) with risk of preeclampsia. Gynecol Endocrinol. (2023) 39:2146089. doi: 10.1080/09513590.2022.2146089 36395814

[41] Liu S. The role of vitamin D receptor gene polymorphisms in gestational diabetes mellitus susceptibility: a meta-analysis. Diabetol Metab Syndr. (2021) 13:144. doi: 10.1186/s13098-021-00764-y 34903261 PMC8670261

[42] Shaat N Katsarou A Shahida B Prasad RB Kristensen K Planck T. Association between the rs1544410 polymorphism in the vitamin D receptor (VDR) gene and insulin secretion after gestational diabetes mellitus. PLoS One. (2020) 15:e0232297. doi: 10.1371/journal.pone.0232297 32407388 PMC7224565

[43] Mo M Shao B Xin X Luo W Si S Jiang W. The association of gene variants in the vitamin D metabolic pathway and its interaction with vitamin D on gestational diabetes mellitus: A prospective cohort study. Nutrients. (2021) 13:4220. doi: 10.3390/nu13124220 34959770 PMC8706628

[44] Ali Khan IA-O Alhaizan MA-O Neyazi SM Al-Hakeem MM Alshammary AA-O. Relevance of serum levels and functional genetic variants in vitamin D receptor gene among Saudi women with gestational diabetes mellitus. Nutrients. (2023) 15:4288. doi: 10.3390/nu15194288 37836571 PMC10574375

[45] Zeng Q Zou D Wei Y Ouyang Y Lao Z Guo R. Association of vitamin D receptor gene rs739837 polymorphism with type 2 diabetes and gestational diabetes mellitus susceptibility: a systematic review and meta-analysis. Eur J Med Res. (2022) 27:65. doi: 10.1186/s40001-022-00688-x 35526059 PMC9080160

[46] Christoph P Challande P Raio L Surbek D. High prevalence of severe vitamin D deficiency during the first trimester in pregnant women in Switzerland and its potential contributions to adverse outcomes in the pregnancy. Swiss Med weekly. (2020) 150:w20238. doi: 10.4414/smw.2020.20238 32502277

[47] Bromage S Enkhmaa D Baatar T Garmaa G Bradwin G Yondonsambuu B. Comparison of seasonal serum 25-hydroxyvitamin D concentrations among pregnant women in Mongolia and Boston. J Steroid Biochem Mol Biol. (2019) 193:105427. doi: 10.1016/j.jsbmb.2019.105427 31323345 PMC11536343

[48] Alzaim M Wood RJ. Vitamin D and gestational diabetes mellitus. Nutr Rev. (2013) 71:158–67. doi: 10.1111/nure.12018 23452283

[49] Giustina A Bouillon R Binkley N Sempos C Adler RA Bollerslev J. Controversies in vitamin D: A statement from the third international conference. JBMR Plus. (2020) 4:e10417. doi: 10.1002/jbm4.10417 33354643 PMC7745884

[50] Tripathi P Rao YK Pandey K Gautam KA. Significance of vitamin D on the susceptibility of gestational diabetes mellitus - A meta-analysis. Indian J Endocrinol Metab. (2019) 23:514–24. doi: 10.4103/ijem.IJEM_184_19 PMC6873259 31803590

[51] Cheng Y Chen J Li T Pei J Fan Y He M. Maternal vitamin D status in early pregnancy and its association with gestational diabetes mellitus in Shanghai: a retrospective cohort study. BMC Pregnancy Childbirth. (2022) 22:819. doi: 10.1186/s12884-022-05149-1 36335302 PMC9636619

[52] Salakos E Rabeony T Courbebaisse M Taieb J Tsatsaris V Guibourdenche J. Relationship between vitamin D status in the first trimester of pregnancy and gestational diabetes mellitus - A nested case-control study. Clin Nutr. (2021) 40:79–86. doi: 10.1016/j.clnu.2020.04.028 32448701

[53] Milajerdi A Abbasi F Mousavi SM Esmaillzadeh A. Maternal vitamin D status and risk of gestational diabetes mellitus: A systematic review and meta-analysis of prospective cohort studies. Clin Nutr. (2021) 40:2576–86. doi: 10.1016/j.clnu.2021.03.037 33933723

[54] ACOG Committee Opinion No. 495. Vitamin D: Screening and supplementation during pregnancy. Obstet Gynecol. (2011) 118:197–8. doi: 10.1097/AOG.0b013e318227f06b 21691184

[55] Holick MF Binkley NC Bischoff-Ferrari HA Gordon CM Hanley DA Heaney RP. Endocrine Society. Evaluation, treatment, and prevention of vitamin D deficiency: an Endocrine Society clinical practice guideline. J Clin Endocrinol Metab. (2011) 96:1911–30. doi: 10.1210/jc.2011-0385 21646368

[56] Zhang Q Cheng Y He M Li T Ma Z Cheng H. Effect of various doses of vitamin D supplementation on pregnant women with gestational diabetes mellitus: A randomized controlled trial. Exp Ther Med. (2016) 12:1889–95. doi: 10.3892/etm.2016.3515 PMC4998009 27588106

[57] Cesareo R Attanasio R Caputo M Castello R Chiodini I Falchetti A. AME and italian AACE chapter. Italian association of clinical endocrinologists (AME) and Italian chapter of the American association of clinical endocrinologists (AACE) position statement: clinical management of vitamin D deficiency in adults. Nutrients. (2018) 10:546. doi: 10.3390/nu10050546 29702603 PMC5986426

[58] Rusińska A Płudowski P Walczak M Borszewska-Kornacka MK Bossowski A Chlebna-Sokół D. et al.Vitamin D Supplementation Guidelines for General Population and Groups at Risk of Vitamin D Deficiency in Poland-Recommendations of the Polish Society of Pediatric Endocrinology and Diabetes and the Expert Panel With Participation of National Specialist Consultants and Representatives of Scientific Societies-2018 Update. Front Endocrinol (Lausanne). (2018) 9:246. doi: 10.3389/fendo.2018.00246 29904370 PMC5990871

[59] Pludowski P Takacs I Boyanov M Belaya Z Diaconu CC Mokhort T. Clinical practice in the prevention, diagnosis and treatment of vitamin D deficiency: A central and eastern European expert consensus statement. Nutrients. (2022) 14:1483. doi: 10.3390/nu14071483 35406098 PMC9002638

[60] Kloppel E. Benefits of vitamin D supplementation on pregnancy of rats with pregestational diabetes and their offspring. Reproductive. (2023) 30:1241–56. doi: 10.1007/s43032-022-01056-0 35999443

[61] Huang S Fu J Zhao R Wang B Zhang M Li L. The effect of combined supplementation with vitamin D and omega-3 fatty acids on blood glucose and blood lipid levels in patients with gestational diabetes. Ann Palliat Med. (2021) 10:5652–8. doi: 10.21037/apm-21-1018 34107720

[62] Merewood A Mehta SD Chen TC Bauchner H Holick MF. Association between vitamin D deficiency and primary cesarean section. J Clin Endocrinol Metab. (2009) 94:940–5. doi: 10.1210/jc.2008-1217 PMC2681281 19106272

[63] van Weert B van den Berg D Hrudey EJ Oostvogels AJJM de Miranda E Vrijkotte TGM. Is first trimester vitamin D status in nulliparous women associated with pregnancy related hypertensive disorders? Midwifery. (2016) 34:117–22. doi: 10.1016/j.midw.2015.12.007 26805604

[64] Augustin H Mulcahy S Schoenmakers I Bullarbo M Glantz A Winkvist A. Late pregnancy vitamin D deficiency is associated with doubled odds of birth asphyxia and emergency caesarean section: A prospective cohort study. Matern Child Health J. (2020) 24:1412–8. doi: 10.1007/s10995-020-02999-z PMC7560912 32844359

[65] Das B Singhal SR Ghalaut VS. Evaluating the association between maternal vitamin D deficiency and preeclampsia among Indian gravidas. Eur J Obstet Gynecol Reprod Biol. (2021) 261:103–9. doi: 10.1016/j.ejogrb.2021.04.014 33915489

[66] Wei SQ Bilodeau JF Julien P Luo ZC Abenhaim HA Bi WG. oxidative stress, and pre-eclampsia. Int J Gynaecol Obstet. (2021) 154:444–50. doi: 10.1002/ijgo.13559 33350462

[67] Nema J Wadhwani N Randhir K Dangat K Pisal H Kadam V. Association of maternal vitamin D status with the risk of preeclampsia. Food Funct. (2023) 14:4859–65. doi: 10.1039/d3fo00007a 37129568

[68] Amiri M Rostami M Sheidaei A Fallahzadeh A Ramezani Tehrani F. Mode of delivery and maternal vitamin D deficiency: an optimized intelligent Bayesian network algorithm analysis of a stratified randomized controlled field trial. Sci Rep. (2023) 13:8682. doi: 10.1038/s41598-023-35838-6 37248326 PMC10226976

[69] Loy SL Lek N Yap F Soh SE Padmapriya N Tan KH. Growing Up in Singapore Towards Healthy Outcomes (GUSTO) study group. Association of Maternal Vitamin D Status with Glucose Tolerance and Caesarean Section in a Multi-Ethnic Asian Cohort: The Growing Up in Singapore Towards Healthy Outcomes Study. PloS One. (2015) 10:e0142239. doi: 10.1371/journal.pone.0142239 26571128 PMC4646602

[70] Gómez-Carrascosa I Sánchez-Ferrer ML Arense-Gonzalo JJ Prieto-Sánchez MT Alfosea-Marhuenda E Iniesta MA. Associations between maternal circulating 25-hydroxyvitamin D concentration and birth outcomes-Mode of delivery and episiotomy rate: A prospective cohort study. Nurs Open. (2021) 8:3645–54. doi: 10.1002/nop2.915 PMC8510719 33991181

[71] Kim KS Park SW Cho YW Kim SK. Vitamin D deficiency at mid-pregnancy is associated with a higher risk of postpartum glucose intolerance in women with gestational diabetes mellitus. Endocrinol Metab (Seoul). (2020) 35:97–105. doi: 10.3803/EnM.2020.35.1.97 32207269 PMC7090297

[72] Lee HJ Han JY Hwang JH. Association between preterm premature rupture of membranes and vitamin D levels in maternal plasma and umbilical cord blood of newborns: A prospective study. Obstet Gynecol. (2022) 49:158. doi: 10.31083/j.ceog4907158

