# Corrigendum: Relationship between vitamin D deficiency and gestational diabetes: a narrative review

**DOI:** 10.3389/fendo.2024.1548353

**Published:** 2025-01-06

**Authors:** Caiqiong Lin, Haiwei Liu

**Affiliations:** Department of Endocrinology, Hainan General Hospital, Hainan Affiliated Hospital of Hainan Medical University, Haikou, Hainan, China

**Keywords:** vitamin D deficiency, vitamin D receptor gene, gestational diabetes mellitus, correlation, dose supplementation

In the published article, there was an error, regarding the duration of treatment.

A correction has been made to section *2.6.3 Correlation between vitamin D dose and gestational diabetes mellitus*. This sentence previously stated:

“A treatment duration of 4–12 weeks is recommended, with the aim to target concentrations of 30 to 50 ng/mL (59).”

The corrected sentence appears below:

“A treatment duration of entire pregnancy and lactation is recommended, with the aim to target concentrations of 30 to 50 ng/mL (59).”

The authors apologize for this error and state that this does not change the scientific conclusions of the article in any way. The original article has been updated.

